# The underlying mechanisms of lorlatinib penetration across the blood*‐*brain barrier and the distribution characteristics of lorlatinib in the brain

**DOI:** 10.1002/cam4.3061

**Published:** 2020-04-28

**Authors:** Wei Chen, Dujia Jin, Yafei Shi, Yujun Zhang, Haiyan Zhou, Guohui Li

**Affiliations:** ^1^ Department of Pharmacy National Cancer Center/National Clinical Research Center for Cancer/Cancer Hospital Chinese Academy of Medical Sciences and Peking Union Medical College Beijing China; ^2^ Institute of Materia Medica Chinese Academy of Medical Sciences and Peking Union Medical College Beijing China

**Keywords:** blood‐brain barrier, Crizotinib, Lorlatinib, SPP1

## Abstract

**Objective:**

To clarify the distribution of lorlatinib in the brain and elucidate the molecular mechanisms of lorlatinib penetration across the blood‐brain barrier (BBB).

**Methods:**

Cytological experiments were performed to investigate the growth inhibitory effect of lorlatinib on different cells (endothelial cells HUVEC, HMEC‐1, and HCMEC/D3) and to investigate the protective effect of lorlatinib on neuronal cells after SH‐SY5Y hypoxia/reoxygenation injury. Furthermore, rat brain tissue was sequenced, and the differentially expressed genes (secreted phosphoprotein 1 (SPP1), vascular endothelial growth factor (VEGF), transforming growth factor beta (TGF‐β), Claudin, ZO‐1 and P‐gp) in several different drug treatment groups were verified by Real‐Time PCR. Lorlatinib brain distribution was predicted by physiologically based pharmacokinetics (PBPK).

**Results:**

Lorlatinib and crizotinib both had inhibitory effects on endothelial cells, however lorlatinib inhibited the growth of HCMEC/D3 more efficaciously than crizotinib. In the SH‐SY5Y hypoxia model, lorlatinib had a greater protective effect on nerve cell damage caused by hypoxia and reoxygenation than crizotinib. The expression of SPP1, VEGF, TGF‐β, and Claudin in brain tissue was significantly downregulated after lorlatinib administration, and the expression level of early growth transcription factor 1 (Egr‐1) was significantly increased. The PBPK model successfully described lorlatinib concentrations in blood and brain tissue in the mouse model and gave a brain tissue partition coefficient of 0.7.

**Conclusion:**

Lorlatinib can increase the permeability of the blood‐brain barrier whereby we suggest its underlying working mechanism is related to downregulating SPP1, inhibiting VEGF, TGF‐β, and Claudin subsequently reducing the number of tight junctions between BBB cells. Lorlatinib plays a protective role on injured nerve cells and does not change the amount of P‐gp expression in brain tissue, which may be important for its ability to be efficacious across the BBB with a low incidence of resistance.

## Graphical abstract

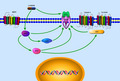

## 1 INTRODUCTION

**Figure 1:**
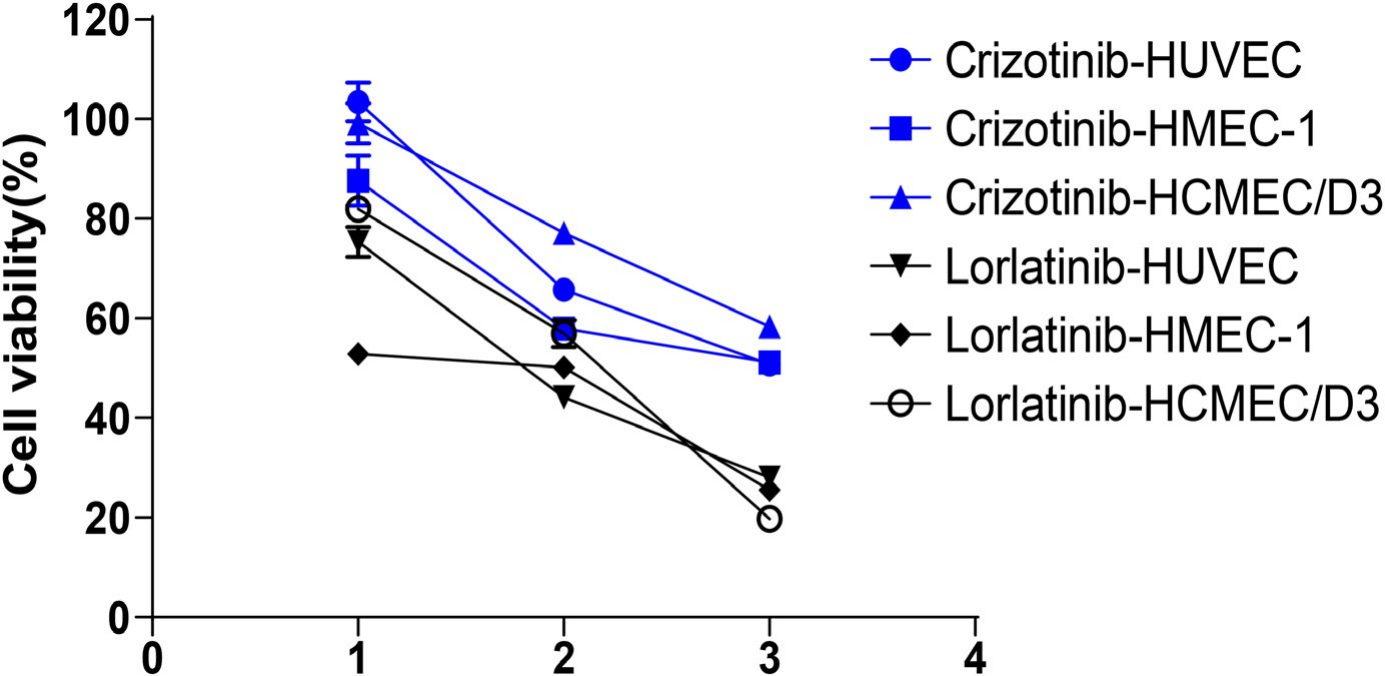
Inhibitive effects on growth of endothelial cells

For several decades, lung cancer has been the most common type of malignant tumor in the world and the number of lung cancer deaths worldwide is expected to continuously and rapidly grow in the future.^1^ According to the available research data, the brain is a preferential metastasis site of lung cancer, with a metastasis frequency of 25%‐40%.^2^, ^3^, ^4^, ^5^, ^6^ Crizotinib and lorlatinib (Figure 1) are second‐ and third‐ generation therapeutic inhibitors targeting anaplastic lymphoma kinase (ALK) and are commonly used drugs for the treatment of lung cancer. When comparing the two, previous investigations have reported that lorlatinib treatment leads to a lower clinical brain metastasis rate and a superior therapeutic effect on brain metastasis.^6^, ^7^, ^8^, ^9^, ^10^ The blood brain barrier (BBB) is a special barrier between the blood circulation and the nerve tissue of the brain. It can effectively restrict certain substances in the blood from entering the brain environment, thus protecting the brain tissue from harmful substances. The blood brain barrier plays an important role in ensuring the stability of the brain tissue environment.^11^, ^12^, ^13^ Cancer cells that metastasize to the brain must first pass through the blood‐brain barrier. Concurrently, a drug therapy for brain metastasis in lung cancer patients must also first pass through the blood‐brain barrier. Therefore, the abnormal opening of the BBB plays a significant role both in the brain metastasis itself, and in the treatment of brain metastasis of lung cancer cells. Crizotinib and lorlatinib both target ALK and have significant inhibitory effects on ALK‐positive lung cancer cells,^14^ however, lorlatinib is better at crossing the blood brain barrier (BBB), improving intracranial disease control.^15^ This suggests that lorlatinib may be able to act upon the blood‐brain barrier, promoting its opening to enhance the therapeutic effect on brain metastasis. The purpose of this study was to investigate whether the BBB is affected by lorlatinib exposure, to clarify what effect lorlatinib has on BBB and to explore its mechanism.

In this study, we found a strong correlation between the abnormally downregulated expression of the SPP1 gene and abnormal opening of BBB, demonstrated the ability of lorlatinib to improve BBB permeability, and identified the molecular mechanisms by which lorlatinib alters BBB cell expression and enables the opening of the BBB. The results of this study provide new ideas for the development of clinical drugs for the brain.

## 2 MATERIALS AND METHODS

### 2.1 Cell lines

HUVEC (human umbilical vein endothelial cells), HMEC‐1 (human microvascular endothelial cells), HCMEC/D3 (immortalized human brain microvascular endothelial cells), SH‐SY5Y (human neuroblastoma cells) were all purchased from Beijing Zhongke Quality Inspection Biotechnology Co., Ltd.

### 2.2 Animals and ethics

All animal‐involved experiments were approved by the Institutional Experimental Animal Ethical Committee. All the animal‐related experimental procedures were carried out strictly in accordance with guidelines of this committee. Eight‐week‐old male SD (certificate number 11401300081802) rats weighing 180‐220 g were purchased from Beijing HFK Bioscience Co., Ltd. A total of 30 rats were used in this study. All rats were housed in standard ventilated cages (2‐4 per cage) under constant temperature, humidity and a 12/12 ‐hour light/dark cycle. All rats were given free access to water and fed with standard laboratory chow during the experiment.

### 2.3 Experimental materials

Lorlatinib (PF‐06463922, CAS No.: 1454846‐35‐5) and crizotinib (PF‐02341066, CAS No.: 877399‐52‐5) were purchased from Med Chem Express (MCE). Evans Blue (batch number: C10154250) was purchased from beyotime Biotechnology Co., Ltd. The EB test kit (batch number: 20180615) was purchased from Beijing solarbio science & technology Co.,Ltd. The CCK8 test kit (batch number 20180824‐2) was purchased from Nanjing Vazyme Biotech Co., Ltd.

### 2.4 Primer synthesis sequence

The designed primer sequences were listed in Table 1.

**Table 1:** Primer synthesis sequence

Gene	Primer Pair (5′‐ 3′) F, forward; R, reverse
Rat β‐actin	F: 3‐GTAAAGACCTCTATGCCAACA‐5 R: 3‐GGACTCATCGTACTCCTGCT‐5
Rat Spp1	F:5‐ AGGTCATCCCAGTTGCC‐3 R: 5‐GGCCCTCTGCTTATACTCC‐3
Rat Vegfa	F: 5‐ACAGGGAAGACAATGGGA −3 R: 5‐CTGGAAGTGAGCCAACG‐3
Rat Tgfb1	F: 5′‐CCTACATTTGGAGCCTGGA −3′ R: 5′‐CCGGGTTGTGTTGGTTG‐3′
Rat Egr1	F: 5′‐CCGAGCGAACAACCCTAC‐3′ R: 5′‐ GGTGATGGGAGGCAACC‐3′
Rat Claudin‐5	F: 5‐ TGAAGGACCCATCTGCCT −3 R: 5′‐TGCTTGCTGTGAGAACTGG −3′
Rat ZO‐1	F: 5′‐CTTTGACCAGTACCCACGA −3′ R: 5′‐TCAGAGGAGGAACAACTGC −3′
Rat P‐gP	F: 3‐ TCGGTTGTGCATGGGTT‐5 R: 3‐GCAGAGGAAAAGGCCAGA‐5

### 2.5 Experimental equipment

Microplate reader (ELX808IU), High speed refrigerated centrifuge (HC‐2518R), Protein nucleic acid spectrophotometer (Eppendorf BioPhotometer plus), real‐time PCR (ABI 7500 fast), Cell and tissue disintegrators (F6/10model), PCR (GE4852).

### 2.6 Experimental method

#### 2.6.1 Inhibitive effects on growth of endothelial cells

The endothelial cell lines HUVEC, HMEC‐1, HCMEC/D3 were cultured in vitro. Lorlatinib and crizotinib inhibitive effects on endothelial cell growth were investigated at dose concentrations of 10, 100, and 1000 μmol/L.

#### 2.6.2 Neuroprotective effects on neuronal hypoxia/reoxygenation injury

An OGD/R model in vitro was built using SY5Y. There were four groups (model group, lorlatinib administration group, crizotinib administration group, and control group) in this experiment. The drug‐administered group was given corresponding drugs of two different concentrations (1, 10 μmol/L) after 4 hours of hypoxia. The cell survival rate was detected using the Cell Counting Kit‐8 (CCK‐8) assay after 24 hours of cell culture.

#### 2.6.3 Group and administration

SD rats were acclimated to the environment for one week and were randomly divided into six groups (n = 5 each group): group 1, control; group 2, borneol group; group 3, lorlatinib single administration group; group 4, lorlatinib repeated administration group; group 5, crizotinib single administration group; group 6, crizotinib repeated administration group. Group 1 rats were intraperitoneally injected with 0.9% physiological saline (0.6 mL/100 g) for seven consecutive days. Group 2 rats were given borneol solution (250 mg/kg, i.p.) for seven consecutive days. Group 3 rats were administered with lorlatinib (7 mg/kg, i.p.) on the seventh day, following six consecutive days of 0.9% physiological saline administration. Group 4 rats were intraperitoneally injected with 7 mg/kg lorlatinib for seven consecutive days. Group 5 rats were administered crizotinib (7 mg/kg, i.p.) on the seventh day, following six consecutive days of 0.9% physiological saline administration. Group 6 rats were intraperitoneally injected with 7mg/kg crizotinib for seven consecutive days.

#### 2.6.4 Effect of lorlatinib on opening of rat BBB

Two rats in each group were randomly selected and 0.5% EB was injected into the tail vein. One hour after injection, the rats were anesthetized with chloral hydrate. After the heart was perfused with saline, the intact brain was taken, and pathological slices were made to detect the degree of blue staining in the brain. The brain pathological slices were homogenized in physiological saline, and the EB content was detected using the EB test kit.

#### 2.6.5 cDNA sequencing

One hour after the last administration, the heart of the remaining three rats in each group was perfused with saline, the intact brain was taken for sequencing and molecular biology research.

The total RNA in the brain was extracted. After the mRNA library of each sample which passed the quality test was constructed, sequencing was performed by the BGI‐Shenzhen. According to the sequencing results, the effective data were selected and the FPKM of each gene in the mRNA library of detected samples was obtained. The differences in gene expression levels between the groups was analyzed according to the data.

#### 2.6.6 Real‐time PCR

The total mRNA in the brain tissue was extracted using the RNA extraction kit. The concentration of each sample was detected, and mRNA was reverse‐transcribed into cDNA. The expression level of each gene was obtained using a real‐time fluorescent quantitative PCR kit.

#### 2.6.7 Lorlatinib brain distribution prediction by physiologically based pharmacokinetics (PBPK)

Parameters required for building PBPK mouse models had been previously collated, analyzed, and integrated. The lorlatinib PBPK model was built by the Simcyp Simulator V17 Release 1, which was used for all predictions in this study. The tissue distributions of lorlatinib in mouse were previously analyzed by liquid chromatography‐mass spectrometry.^16^ Finally, the lorlatinib brain distribution in mouse predicted by the PBPK model was compared with experimental observations available in previous studies.

## 3 RESULTS

### 3.1 Inhibitive effects on growth of endothelial cells

There is a linear relationship between drug concentration and inhibition rate. Lorlatinib and crizotinib show a significant increase in inhibition rate in HCMEC/D3 with increasing drug concentration and have a relatively gentle increase in inhibition rate in HUVEC and HMEC‐1 with increasing drug concentration, as shown in Figure 1.

### 3.2 Neuroprotective effects of lorlatinib and crizotinib on hypoxia injury in SY5Y cells

**Figure 2:**
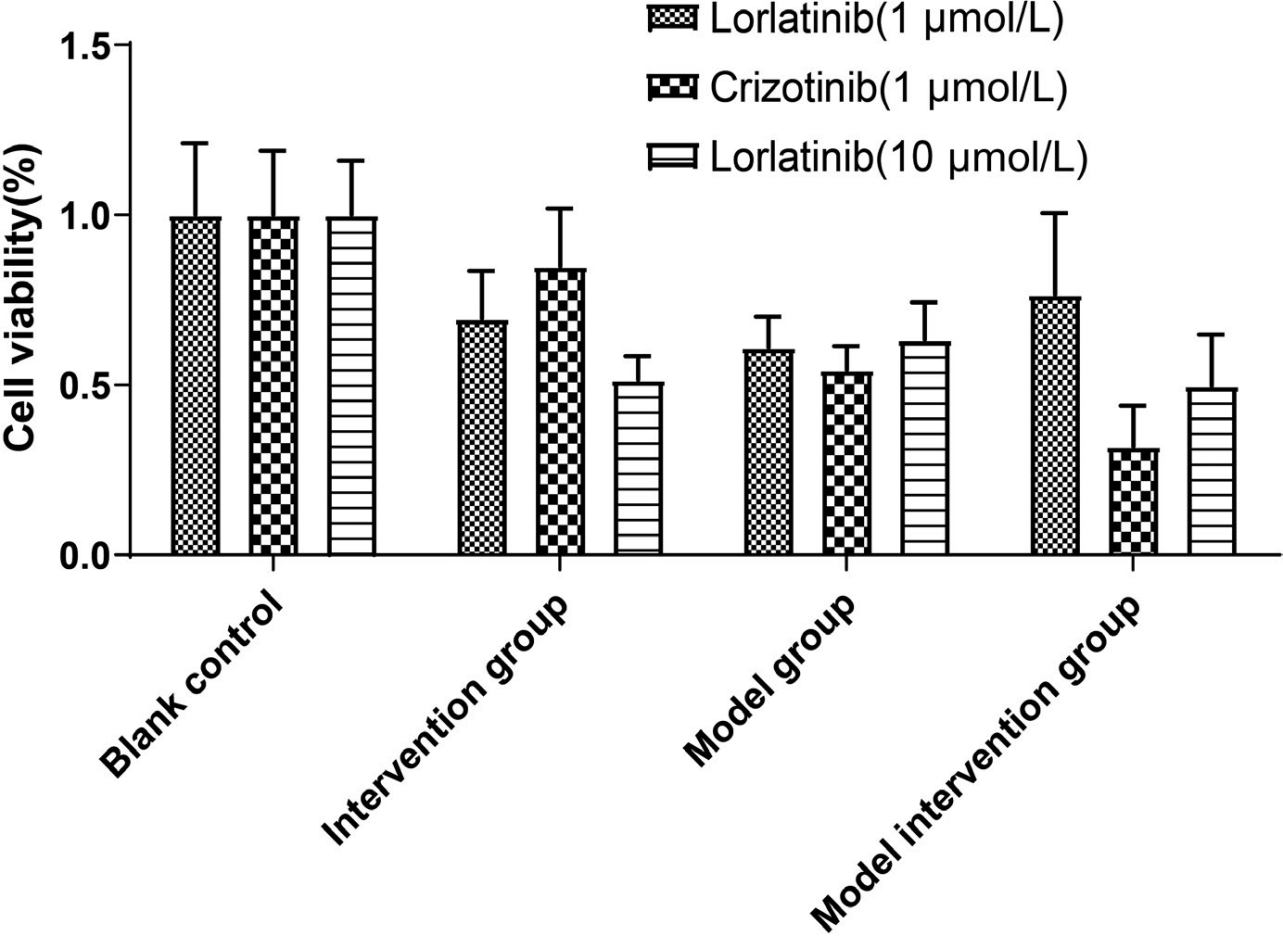
Neuroprotective effects of lorlatinib and crizotinib on hypoxia injury in SY5Y cells

As shown in Figure 2, lorlatinib had a damaging effect on normal cells. Compared with a blank control, the viability of cells treated with 10 μmol/L lorlatinib in the intervention group was 51.5%, whereas after hypoxia‐reoxygenation in the model group, cell viability was 63.3%. Therefore, damage caused by 10 μmol/L lorlatinib to normal cells was more substantial than the level of damage caused to the model‐induced cells. After hypoxia‐reoxygenation injury, 1 μmol/L lorlatinib could significantly increase cell viability from 61.0% to 76.6% indicating that lorlatinib has a protective effect on neuronal injury induced by hypoxia and reoxygenation. However, when the concentration of lorlatinib treatment was increased to 10 μmol/L, it led to a decrease in cell viability to 49.7%. Cell viability was 69.5% vs 84.9% after administration of 1 μmol/L lorlatinib or 1 μmol/L Crizotinib, respectively, in the intervention group, indicating that crizotinib at 1 μmol/L caused less damage to normal cells than lorlatinib at the same concentration. After hypoxia‐reoxygenation injury, the viability of cells after administration of 1 μmol/L Crizotinib was 32.0%, which was reduced compared to the 54.0% viability of untreated cells. Therefore, crizotinib at 1 μmol/L did not display cellular protective effects after hypoxia‐reoxygenation injury.

### 3.3 Effect on blood brain barrier (BBB) permeability

**Figure 3:**
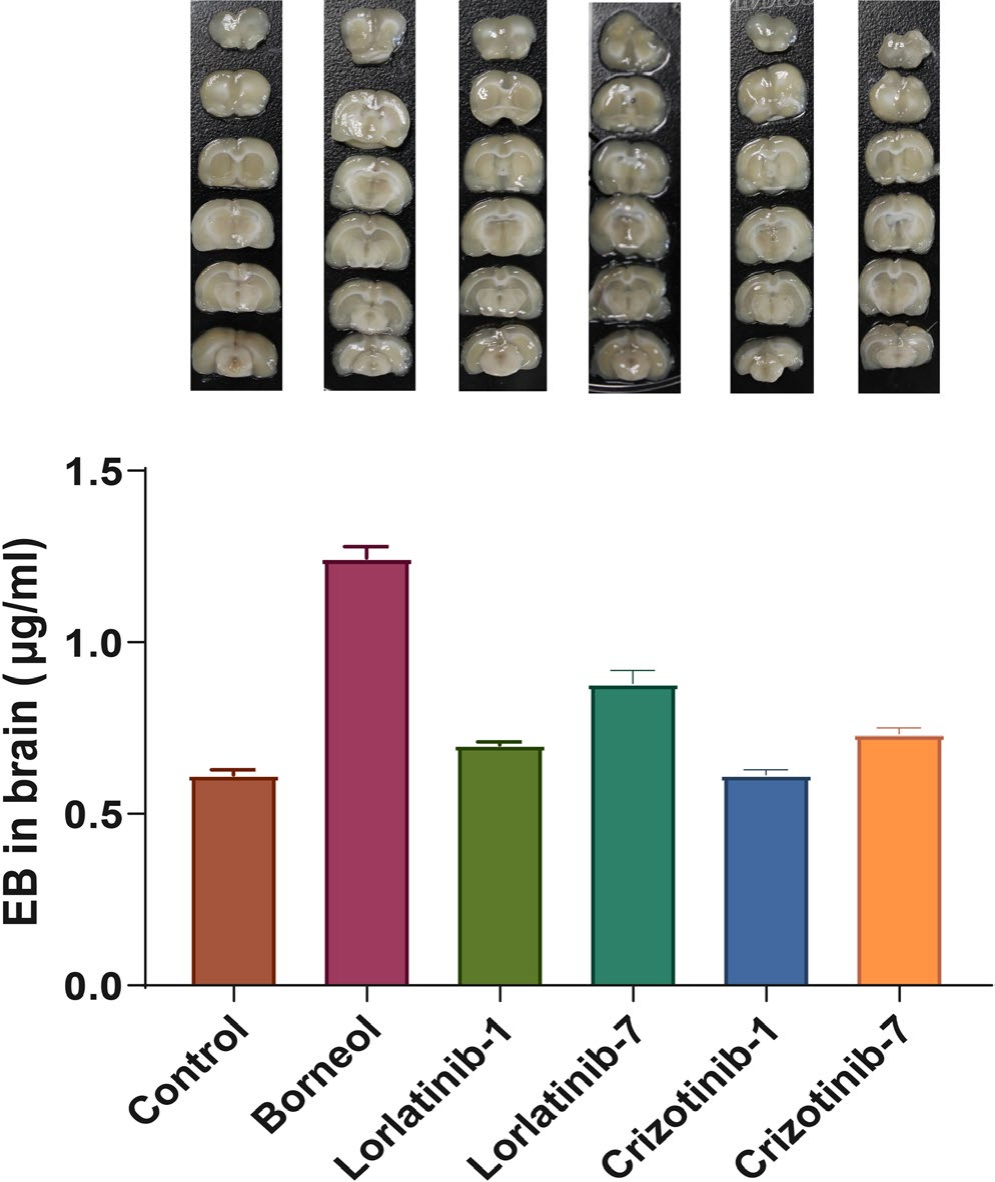
Effect on blood brain barrier (BBB) permeability

As shown in Figure 3, group 2 (borneol group) has the highest brain content of Evans blue among all six groups. Group 4 (lorlatinib repeated administration group) ranked second in Evans blue content. The Evans blue content in the brains of animals in Group 4 was significantly higher than the content of those in Group 3 (lorlatinib single administration group), and also higher than the crizotinib administration groups.

### 3.4 Analysis of sequencing results

**Figure 4:**
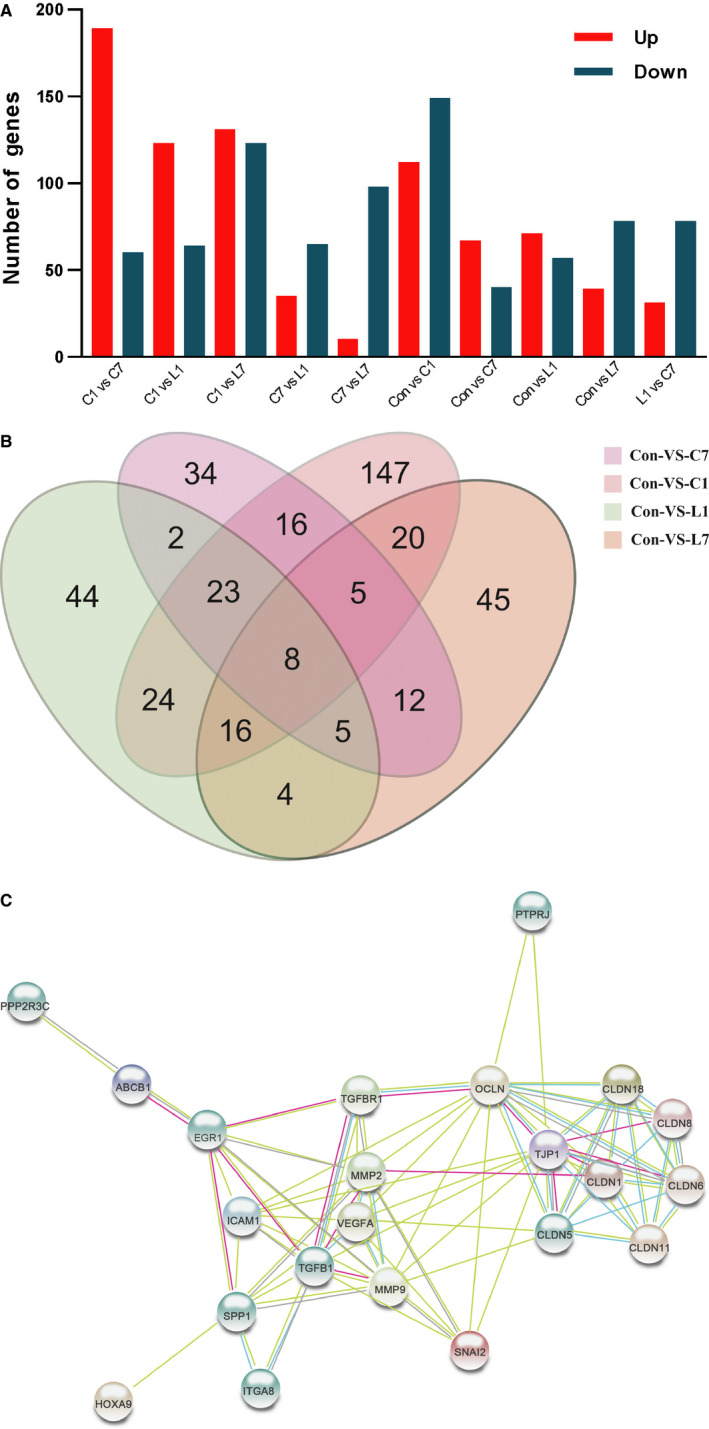
A, The number of differentially expressed genes in each group; B, Sequencing results of each administration group compared with that of the control group; C, The interaction pathway and target of SPP1 and the blood‐brain barrier. (C1: crizotinib single administration; C7: crizotinib repeated administration for 7 consecutive days; L1: lorlatinib single administration; L7: lorlatinib repeated administration for seven consecutive days; Con: control)

The sequencing results are shown in Figure 4. The number of differentially expressed genes in each group is shown in Figure 4A. Sequencing results of each administration group were compared with that of the control group, as shown in Figure 4B. A total of eight genes had differences in expression during comparison, the differential gene expression levels were listed in Table 1. Further screening of eight genes was performed to identify the key gene involved in the increasing of blood‐brain barrier permeability. The screening principle was based on identification of significant differences between the groups, especially the difference between the lorlatinib and crizotinib administration groups. Referring to the relevant literature, there is only one gene, SPP1, that is associated with the blood‐brain barrier among all the eligible genes. The relationship between SPP1 and the blood‐brain barrier was investigated using network pharmacology. The interaction pathway and targets of SPP1 related to the blood‐brain barrier are shown in Figure 4C. Table 2 shows the differential gene expression levels in the brain tissue of rats in each group after administration.

**Table 2:** The differential genes in brain tissue of rats in each group after lorlatinib and crizotinib administration

	Expression difference			
Gene	L1/Con	C7/Con	C1/Con	L7/Con
Cx3cr1	2.190272	2.454761	2.476866	2.118008
Spp1	15.66788	8.039458	28.74617	2.703787
Dcn	2.468665	2.142474	2.29958	2.710884
Dio3	2.107408	2.584075	2.57212	2.524891
Htra4	5.465026	5.472599	4.807308	3.602364
Fibcd1	2.450375	3.289535	2.458418	3.089773
Fam129c	2.294355	2.137241	3.218726	2.806141
Cbln4	3.652828	2.875163	4.228116	3.993962

### 3.5 qPCR results

**Figure 5:**
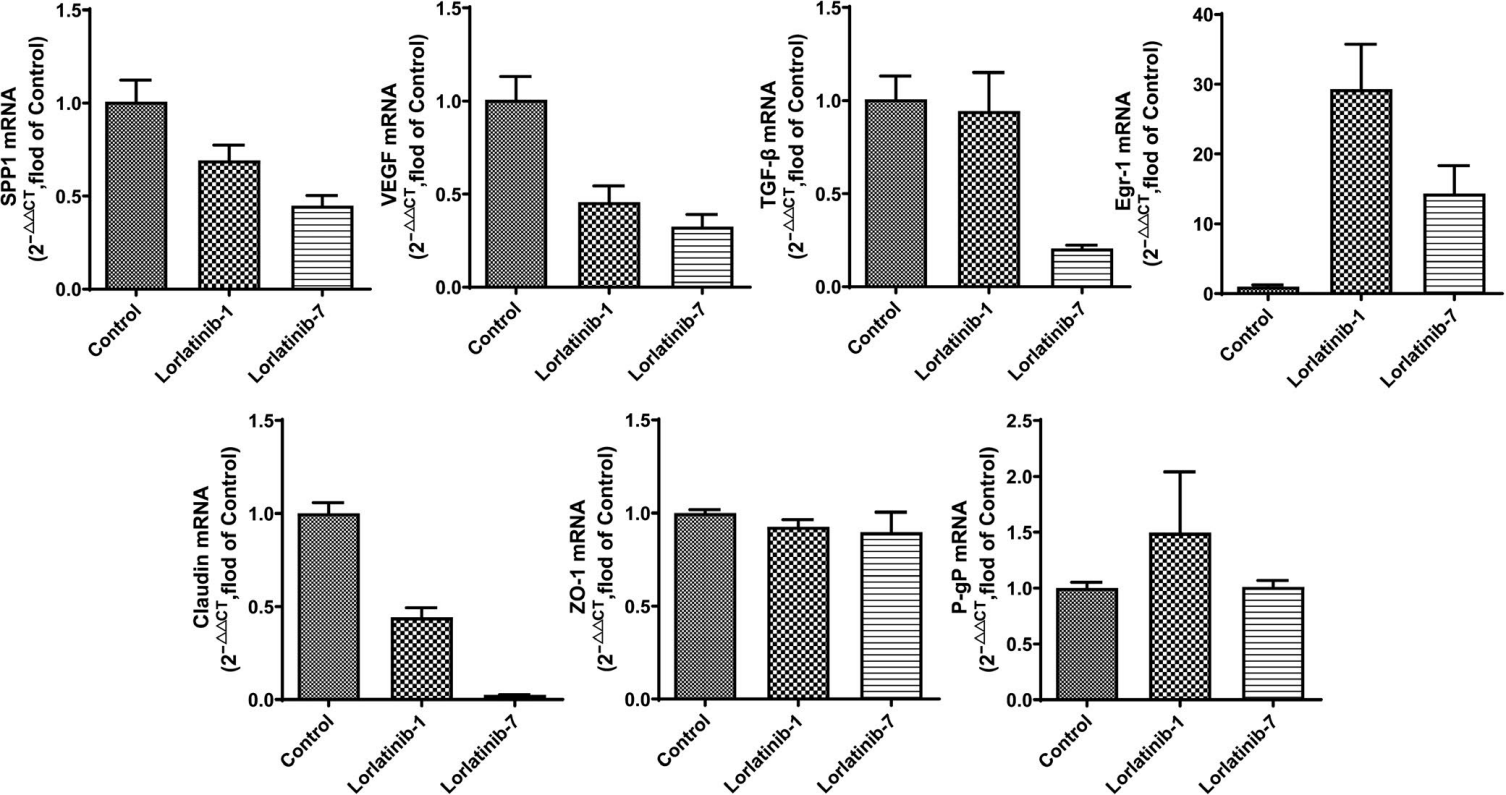
qPCR results of BBB‐related genes under the action of lorlatinib

**Figure 6:**
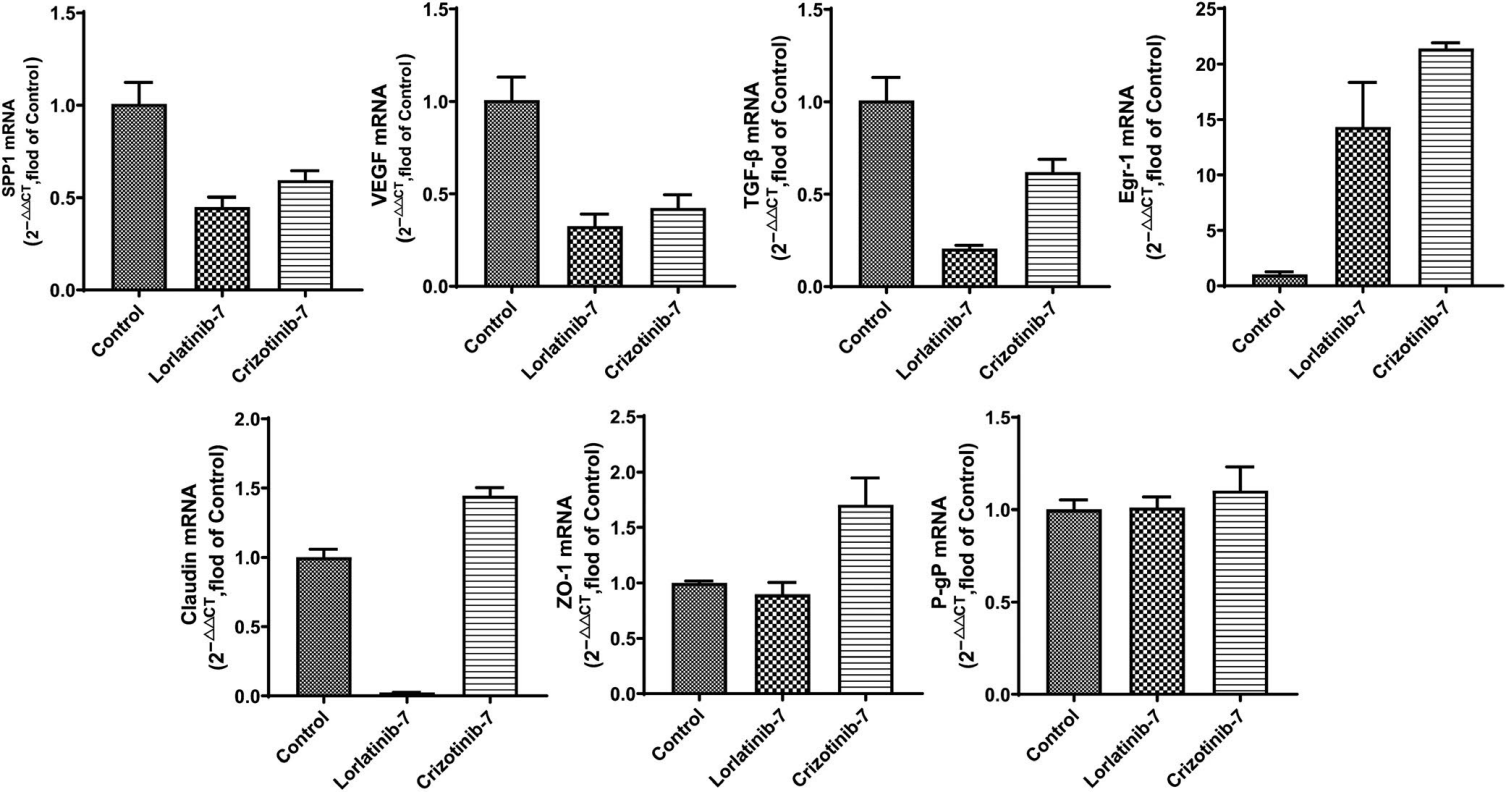
qPCR results of BBB‐related genes under the action of lorlatinib and crizotinib

As shown in Figure 5, it was found that the mRNA expression of SPP1 (0.45 ± 0.05 vs 0.69 ± 0.08), TGF‐β (0.21 ± 0.02 vs 0.94 ± 0.21), Egr‐1 (14.32 ± 4.02 vs 29.29 ± 6.44) and Claudin (0.02 ± 0.00 vs 0.44 ± 0.05) in brain tissue after repeated administration of lorlatinib was significantly lower than after single administration. There was no significant difference in the mRNA expression of ZO‐1 and VEGF between groups. As shown in Figure 6, repeated administration of lorlatinib led to a significant reduction in the mRNA expression of SPP1 (0.45 ± 0.05 vs 0.60 ± 0.05), TGF‐β (0.21 ± 0.02 vs 0.62 ± 0.07), Egr‐1 (14.32 ± 4.02 vs 21.39 ± 0.54), ZO‐1 (0.90 ± 0.11 vs 1.70 ± 0.24) and Claudin (0.02 ± 0.00 vs 1.45 ± 0.06) in brain tissue, compared to after repeated administration of crizotinib. Repeated administration of lorlatinib had no effect on P‐gp expression, whereas P‐gp expression increased slightly after repeated crizotinib administration compared with the control.

### 3.6 Lorlatinib brain distribution prediction by Physiologically Based Pharmacokinetics (PBPK)

**Figure 7:**
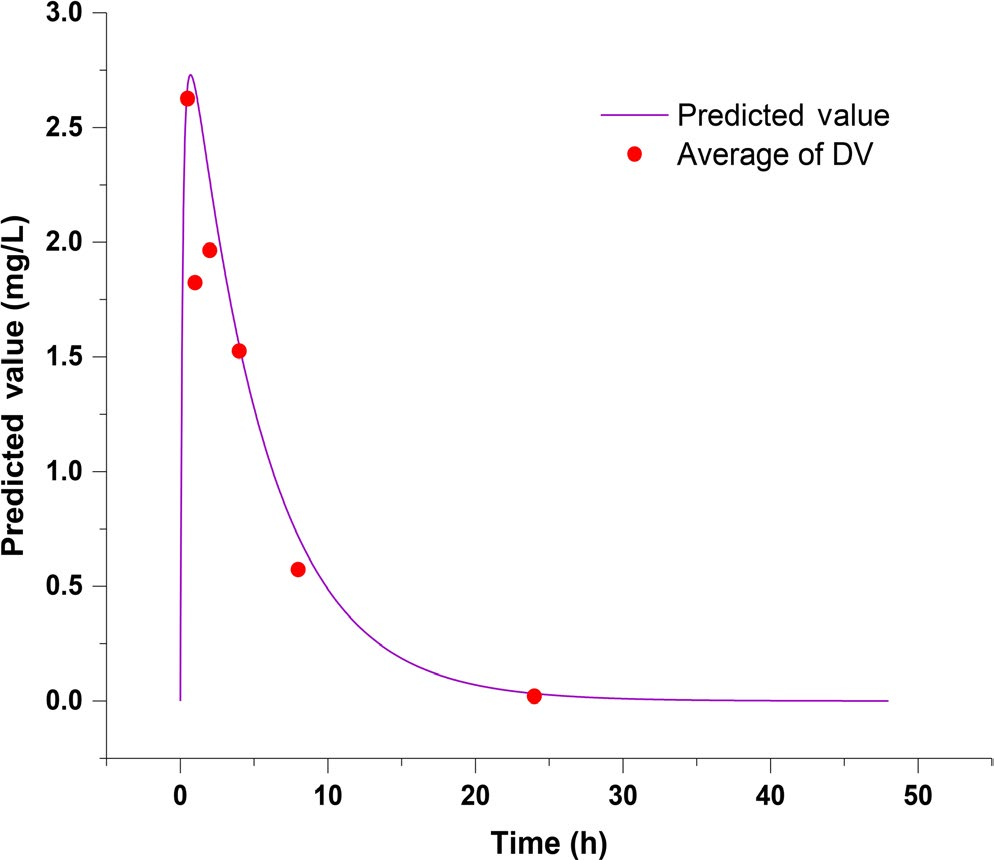
Concentration of lorlatinib in blood

**Figure 8:**
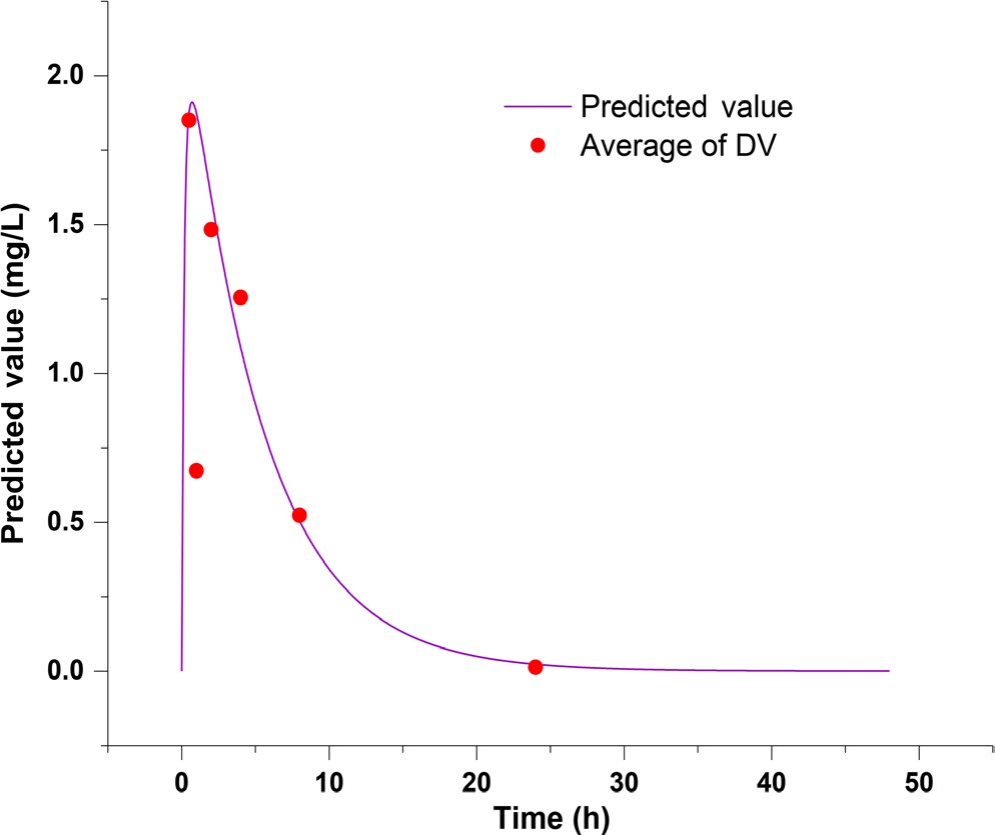
Concentration of lorlatinib in brain

A Physiologically Based Pharmacokinetics (PBPK) model was successfully established by Simcyp Mouse Version 18 Release 1. Based on the model, the concentration of lorlatinib in the blood and brain of mice were predicted precisely (as shown in Figure 7 and Figure 8). According to the model, the brain tissue partition coefficient was 0.7, and Lorlatinib achieved a maximum blood concentration of 2.73 mg/L at 0.69 hours after administration. Total drug concentration in blood over time (AUC) was 16.7mg/L·h. The peak concentration of 1.91 mg/L in the brain occurred at 0.72 hours after administration, which is slightly later than the peak time of the blood concentration. The AUC of lorlatinib in the brain was 11.7 mg/L·h.

## 4 DISCUSSION

The brain metastasis of lung cancer is linked to both the invasiveness of lung cancer cells and an abnormally permeable BBB in some patients.^17^ Both crizotinib and lorlatinib are drugs targeting the cancer‐driven gene ALK.^18^ Lorlatinib, a third‐generation ALK inhibitor, has shown significant efficacy on BM in previous studies.^19^, ^20^ A well‐structured and functional blood‐brain barrier can effectively block brain metastasis, and downregulation of proteins preserving structural tissue integrity caused by invading tumor cells may lead to brain metastasis.^21^ The inhibitory effect of lorlatinib on lung cancer brain metastasis may be due to its protective effect on the BBB, or may be due to the inhibition of metastasis and proliferation of cancer cells, enabled by the drug entering the brain tissue through the BBB.

It was hypothesized that lorlatinib, in addition to acting on cancer cells, also has an effect on the blood‐brain barrier structure. In this study, lorlatinib administration resulted in an increase in EB content in rat brain tissue, while crizotinib did not show this effect. The result is consistent with the clinical manifestations, indicating that lorlatinib can make the blood‐brain barrier more permeable when compared with crizotinib. Due to the structural characteristics of the blood‐brain barrier, in order to clarify lorlatinib's role in increasing BBB permeability, we observed the effects of lorlatinib on endothelial cells HUVEC, HMEC‐1, HCMEC/D3 respectively. The protective effect of lorlatinib on nerve cells after SH‐SY5Y hypoxia/reoxygenation injury was investigated. It was found that lorlatinib had an effect on BBB cells and nerve cells. In order to further clarify the molecular mechanism of lorlatinib in increasing BBB permeability, we collected the brain tissue for gene sequencing and real‐time PCR detection. The results showed that SPP1, VEGF, TGF‐β, and Claudin genes are downregulated and strongly correlated with abnormal BBB permeability. Combined with network pharmacology, the above experimental results were analyzed to confirm that lorlatinib can downregulate the expression of SPP1 in endothelial cells. On the basis of this we summarize that VEGF, TGF‐β, and Claudin were likely further inhibited and that tight junction dysfunction serves as a key factor, permitting the permeation of drugs across the BBB.

There are drug efflux transporters such as P‐gp on the blood‐brain barrier, and the efflux of crizotinib by P‐gp has been experimentally verified.^22^, ^23^ The upregulation of P‐gp after crizotinib administration is a proven mechanism of crizotinib resistance.^22^, ^24^ In this study, lorlatinib does not cause P‐gp expression to increase in rat brain tissue, which indicates that lorlatinib is not isolated from the brain by P‐gp and will not lose the opportunity to inhibit cancer cell growth in brain tissue due to drug efflux. Although the P‐gp expression after crizotinib administration increased slightly compared with the control, there was no statistically significant difference identified.

The osteopontin (OPN) expressed by the SPP1 gene is a neuroprotective glycoprotein which plays an important role in the maintenance of BBB structure and the recovery of damaged BBB.^25^, ^26^, ^27^ In this study, lorlatinib caused a significant downregulation of the SPP1 gene in rat brain tissue, and real‐time PCR also showed a decrease in OPN content in rat brain tissue. The downregulation of expression of SPP1 caused by lorlatinib administration may be an important finding to consider in the design of drugs with improved BBB permeability.

**Figure 9:**
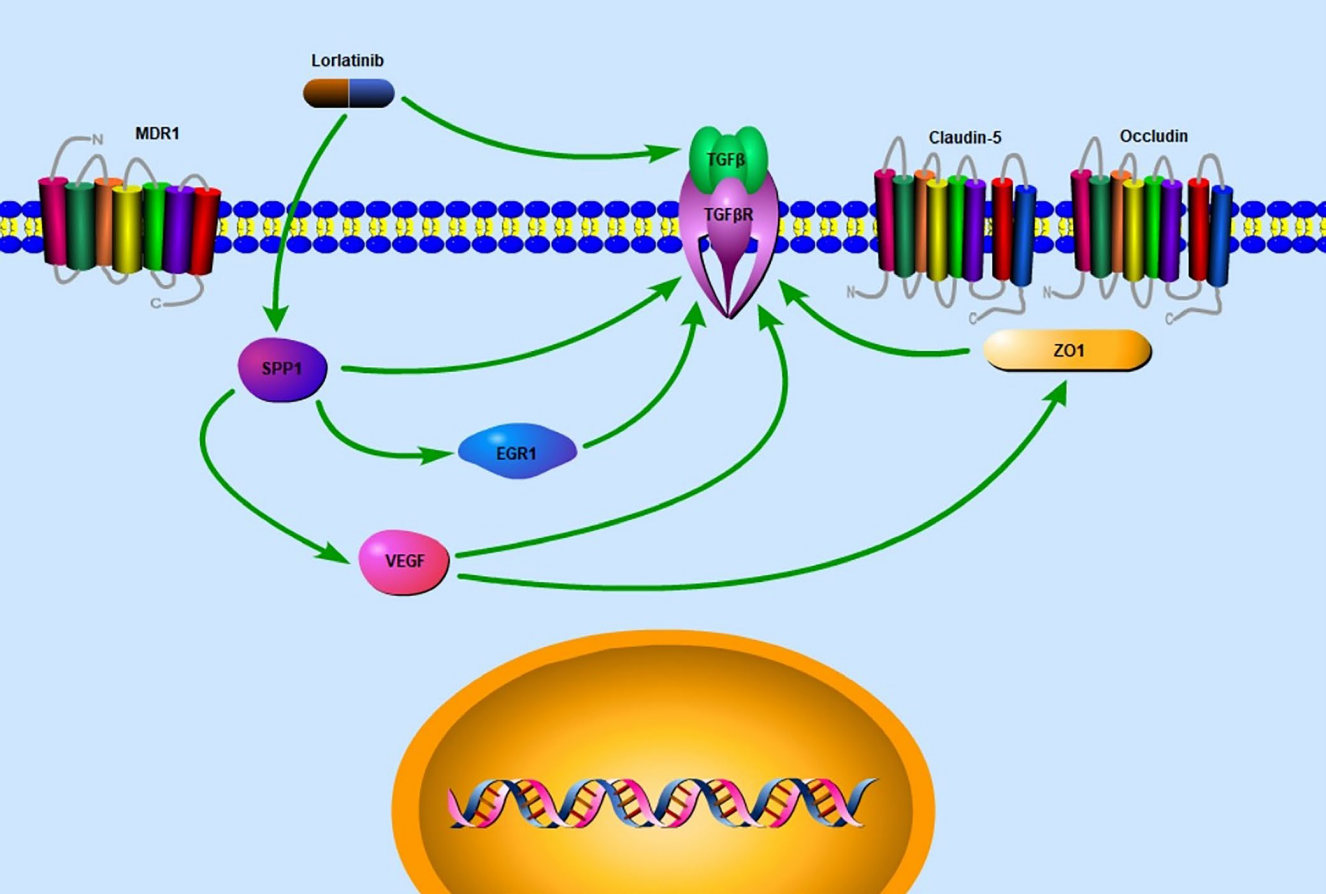
The underlying mechanisms of lorlatinib penetration across the blood‐brain barrier

Based on the experimental results of this study, combined with network pharmacology, we mapped the following mechanism of lorlatinib promoting the permeability of the blood‐brain barrier of the rat (Figure 9). Lorlatinib inhibits the expression of the SPP1 gene in BBB cells, which leads to downregulation of VEGF and TGF‐β and promotes the expression of Egr‐1. This ultimately leads to the destruction of tight junctions between BBB endothelial cells, leading to increased permeability of the BBB. Due to the above effects, lorlatinib subsequently enters the brain tissue through the BBB to inhibit tumor growth and clear the brain of cancerous cells.

The blood‐brain barrier can restrict foreign bodies from entering the central nervous system, which makes it difficult for many therapeutic drugs to penetrate the blood‐brain barrier and produce a cerebrally therapeutic effect. In conclusion, this study provides the first evidence that lorlatinib inhibiting the expression of the SPP1 gene plays a vital role in the underlying mechanisms of lorlatinib penetration across the blood‐brain barrier. Clarifying the transport mechanism of lorlatinib‐promoted penetration will provide new ideas for the design of drugs; improving their central transport and helping reduce the occurrence of drug resistance in the brain could lead to more effective therapeutics.

## CONFLICT OF INTEREST

There are no conflicting interests.

## AUTHOR CONTRIBUTION

Guohui Li and Wei Chen conceived of the study and drafted the manuscript; Wei Chen, Dujia Jin, and Yafei Shi carried out experiments; Wei Chen and Guohui Li analyzed experimental results. Yujun Zhang and Haiyan Zhou assisted with animal experiments. All authors read and approved the final manuscript.

## Data Availability

The data that support the findings of this study are available from the corresponding author upon reasonable request.
